# Investigating the Relationship Between Psoriatic Arthritis and Peyronie’s Disease: A Case Study Analysis

**DOI:** 10.7759/cureus.106962

**Published:** 2026-04-13

**Authors:** Michael J Evans, Vincent J Schmidt, Benjamin Kossman, Dylan Hafer, Curtis King

**Affiliations:** 1 College of Osteopathic Medicine, Kansas City University, Joplin, USA; 2 Department of Primary Care, Kansas City University, Joplin, USA

**Keywords:** case report, chronic inflammation, fibrosis, peyronie’s disease, psoriatic arthritis, social determinants of health

## Abstract

The relationship between psoriatic arthritis (PsA) and Peyronie’s disease (PD) remains poorly understood. PsA is a chronic immune-mediated inflammatory disease, whereas PD is a fibroproliferative disorder of the penile tunica albuginea that leads to penile curvature, pain, and sexual dysfunction. Both conditions involve chronic inflammation and dysregulated tissue remodeling. We report the case of a 42-year-old man with long-standing PsA who developed acute inflammatory PD shortly after discontinuing biologic therapy because of loss of insurance and medication nonadherence. The patient presented with new-onset penile pain and a palpable dorsal plaque in the setting of uncontrolled psoriatic disease. Penile ultrasound demonstrated fibrous plaque formation with linear calcifications consistent with early PD. Management included oral pentoxifylline, topical nitroglycerin, and resumption of systemic immunosuppression with secukinumab through a patient-assistance program, resulting in improvement in both penile symptoms and psoriatic disease activity. To contextualize this presentation, we performed a narrative review of the literature examining reported associations between PD and autoimmune or fibroinflammatory conditions. Prior studies have reported heterogeneous epidemiologic findings, with some suggesting an increased prevalence of psoriasis and PsA among men with PD, while others support a shared profibrotic biology mediated by transforming growth factor-β rather than by classic autoimmunity. This case highlights a potential temporal and pathophysiologic association between uncontrolled systemic inflammation and localized penile fibrosis and underscores the impact of social determinants of health on disease progression. Awareness of this possible association may prompt earlier urologic evaluation in patients with inflammatory arthritis and emphasizes the importance of maintaining continuity of biologic therapy in chronic immune-mediated disease.

## Introduction

Psoriatic arthritis (PsA) is a chronic inflammatory arthropathy affecting approximately 0.25% of the general population and up to 30% of individuals with psoriasis, though prevalence estimates vary considerably depending on diagnostic definitions [1]. While its musculoskeletal and cutaneous manifestations are well characterized, potential associations with other inflammatory or fibrotic conditions remain less defined. Peyronie's disease (PD) affects between 0.4% and 20% of men and involves fibrous plaque formation in the tunica albuginea of the penis, resulting in penile curvature, pain, and erectile dysfunction (ED) [2]. Emerging evidence suggests that chronic inflammatory states may contribute to PD pathogenesis. While PD is primarily mediated by transforming growth factor-β (TGF-β) and aberrant wound healing, PsA is driven by interleukin-23 (IL-23) and Th17-related pathways [3,4].

These overlapping immune mechanisms may link systemic inflammation with the localized fibrotic response observed in these conditions. Understanding a potential association between PsA and PD carries important clinical implications, including earlier recognition of urologic symptoms in patients with systemic inflammatory disease and optimizing disease control to mitigate fibrotic complications. We present a patient with long-standing PsA who developed PD shortly after discontinuing biologic therapy. This case highlights potential inflammatory crosstalk and illustrates how social determinants of health can exacerbate disease progression.

## Case presentation

Patient history and initial presentation

A 42-year-old Caucasian man with a 20-year history of PsA presented to the emergency department for lower back and flank pain. Uncontrolled psoriatic disease and medication nonadherence were noted. His PsA had previously been well controlled on adalimumab (Humira). Still, prolonged opioid use for chronic pain resulted in substance dependence, job loss, and loss of insurance coverage during a divorce. Consequently, he discontinued biologic therapy approximately six months prior to presentation, with progressive worsening of psoriatic symptoms.

Physical examination revealed widespread psoriatic plaques. Laboratory studies and lumbar radiography were unremarkable. Lumbar radiography was also unremarkable. He was discharged with pain control and referrals to community health resources for ongoing management.

Approximately two weeks later, he presented to the community clinic reporting new-onset penile pain and a palpable lump on the dorsal penile shaft. He denied trauma or prior penile symptoms. Examination revealed a firm, nontender plaque on the dorsal aspect of the penis, with active psoriatic lesions similar to those noted for the previous exam.

Diagnostic evaluation

**Figure 1:**
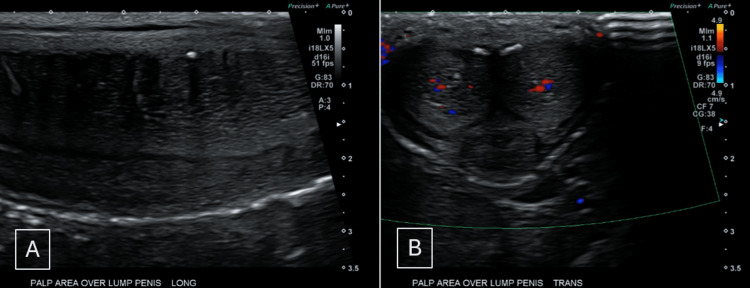
Penile ultrasound findings (A) Long-axis view demonstrating linear echogenic calcifications along the anterior medial corpus cavernosum corresponding to the palpable penile prominence. (B) Transverse view confirming focal tunical calcifications without a discrete mass, consistent with early Peyronie’s disease

Initial laboratory workup was within normal limits, including screening for sexually transmitted infections. Penile ultrasound a few weeks later showed a fibrous plaque with linear calcifications along the anterior-medial aspect of the corpora cavernosa, consistent with acute inflammatory PD (Figure 1). The testes and epididymides appeared normal, with preserved vascularity.

Management and outcome

PsA management was resumed through a patient-assistance program providing secukinumab, an IL-17A inhibitor. He was also started on oral pentoxifylline, topical nitroglycerin ointment, and behavioral modifications, including avoidance of penile trauma, as well as serial photographic monitoring of curvature. Concurrently, he began buprenorphine/naloxone (Suboxone) therapy at an addiction recovery center for opioid dependence.

Although a urology consultation was arranged, subsequent records were unavailable. At follow-up a few months later, the patient demonstrated improved penile curvature with residual mild deformity and no associated pain. His psoriatic lesions had resolved to a large extent, and arthritis was well controlled with continued systemic immunosuppression. The patient was subsequently lost to follow-up and did not return to the community clinic. Written informed consent was obtained from the patient for publication of this case report and accompanying clinical details.

## Discussion

Literature review

Search Strategy and Results

We conducted a narrative review to explore the potential relationship between PsA and PD. A literature search was performed using PubMed, Embase, and Google Scholar databases. Search terms included combinations of “Peyronie’s disease”, “psoriatic arthritis”, “autoimmune disease”, and “fibrosis” to identify relevant studies, case series, and reports describing inflammatory or autoimmune associations with PD. This review was designed to provide a broad, hypothesis-generating overview rather than a systematic review or meta-analysis.

Evidence for PsA-PD association and conflicting epidemiologic findings

Clinical Studies

There has been growing interest in understanding the potential associations between PD and systemic autoimmune conditions, including PsA. Ventimiglia et al. conducted a large multicenter clinical study evaluating autoimmune disease prevalence in men with PD and found autoimmune disorders to be highly comorbid within this population [5]. Notably, psoriasis, PsA, and rheumatoid arthritis were each significantly more common among men with PD than in controls (all p ≤ 0.03), directly isolating PsA as a distinct and significant association. It was also observed that inflammation plays a key role in the sequence of events leading to penile plaque formation, and it was proposed that shared immunogenetic mechanisms, particularly human leukocyte antigen (HLA)-B27 positivity and TGF-β1-mediated fibrosis, may underlie both PsA and PD. Although causation cannot be established, these data strengthen the hypothesis that systemic autoimmune dysregulation may predispose to localized fibrotic responses within the penile tunica albuginea.

In contrast, a large U.S. claims analysis by Pastuszak et al. found no increased risk of subsequent autoimmune diagnoses after PD, despite prior reports suggesting autoimmune comorbidity [6]. Instead, men with PD had a higher risk of benign prostatic hyperplasia (BPH), prostatitis, and lower urinary tract symptoms (LUTS; all HRs ~1.10-1.21) compared with both age-matched healthy controls and men with ED, and an elevated risk of keloids. These findings were interpreted as reflecting a shared fibrotic biology mediated by TGF-β1 and myofibroblast proliferation, rather than an autoimmune diathesis. Taken together with Ventimiglia’s conclusions, the epidemiologic literature remains heterogeneous, with signals supporting both autoimmune overlap and fibroinflammatory comorbidity depending on study design and outcomes assessed [5,6].

Case Reports

Several reports describe PD co-occurring with systemic autoimmune or connective-tissue disorders, as seen in Table 1 [7-14]. Several common themes observed in these reports, such as pathways involving TGF-β, fibroblast activation, and aberrant wound healing, support a plausible association between autoimmune or fibroinflammatory disorders and PD. However, the literature is limited to most case reports and remains susceptible to publication bias and a lack of mechanistic data.

**Table 1:** Reported autoimmune and fibrotic comorbidities associated with Peyronie’s disease

Condition	Study
Sjögren’s syndrome	Kobak and Saraçoğlu [7]
Cogan syndrome	Ollivaud et al. [8]
Primary sclerosing cholangitis + ulcerative colitis	Viteri et al. [9]
Systemic sclerosis	Ordi et al. [10]
Scleroderma	Chen et al. [11]
Paget’s disease of bone	Lyles et al. [12]
Retroperitoneal fibrosis	Akbal et al. [13]
Polyfibromatosis + interstitial granulomatous dermatitis with arthritis	Chen et al. [14]

Pathophysiological considerations

PD Mechanisms

PD pathophysiology involves complex interactions between inflammatory mediators and fibrotic processes. Key mechanisms include excessive TGF-β signaling and collagen deposition. Early disease stages involve the recruitment of inflammatory cells and the production of local cytokines. Later in the progression of disease, disrupted healing responses following microtrauma lead to excessive scar formation. This is exacerbated by an imbalance between matrix metalloproteinases and tissue inhibitors of metalloproteinases, leading to the development of clinically evident PD [3,15,16].

PsA Immunopathology

PsA involves distinct immunologic pathways that drive inflammation, synovitis, and progressive joint damage. Activation of the IL-23/Th17 Axis leads to overproduction of cytokines in genetically predisposed individuals, particularly those with HLA-B27 and non-HLA variants such as IL12B, IL23R, TRAF3IP2, and TYK2. Autoimmune components are known to play a greater role in PsA than in PD, including oligoclonal CD8⁺ T-cell expansion and disease-specific autoantibodies. This further supports the role that dysregulation of adaptive immunity plays in the pathogenesis of PsA [4,17,18].

Potential Mechanistic Links

Both conditions involve inflammatory processes. They differ in that PD is predominantly mediated by TGF-β-driven fibrosis, whereas PsA involves IL-23/Th17-driven inflammation. Potential connecting mechanisms include systemic inflammation, vascular dysfunction, oxidative stress, and connective-tissue abnormalities. Chronic inflammation, seen in both PD and PsA, may predispose to aberrant wound healing and fibrosis, and the common inflammatory pathways involved in disease progression lead to the production of reactive oxygen species and increased oxidative stress. Additionally, both conditions involve altered microcirculation and endothelial dysfunction, leading to vascular compromise and increased susceptibility to connective-tissue disorders.

Supporting these mechanistic links, the role of HLA-B27 was highlighted, carried by approximately half of patients with PsA, and shown in prior studies to be associated with PD [18]. This genetic overlap suggests a shared susceptibility locus that may influence aberrant immune activation and tissue-specific fibrosis. This suggests shared genetic susceptibility that may influence immune activation and tissue-specific fibrosis [5]. In addition to genetic susceptibility, both conditions exhibit upregulation of TGF-β1 and may also demonstrate the Koebner phenomenon, in which minor trauma triggers localized inflammation and scarring [5]. Collectively, these findings provide a biologic rationale for the observed coexistence of PsA and PD in specific individuals.

Complementing these observations, claim-based data indicate that PD clusters with fibroproliferative urologic conditions (BPH, prostatitis, LUTS) and keloids, consistent with a TGF-β1-driven pathway [6]. These findings support a model in which systemic inflammation and local biomechanical triggers converge on profibrotic signaling. The resulting organ-specific phenotypes (penile plaque, prostatic stromal fibrosis, keloid formation) reflect tissue-specific responses to this dual insult. Importantly, this fibrotic remodeling may occur with or without classical autoimmune features, suggesting that chronic inflammation represents one of multiple potential contributors to PD pathogenesis [6]. While the presented case suggests a temporal association, broader conclusions regarding pathophysiology are derived from the existing literature and should be interpreted accordingly.

Limitations and future directions

This case report has inherent limitations and cannot establish causality between PsA and PD based on a single patient's observation. The observed temporal association may also be coincidental, and the concurrent use of multiple therapies (pentoxifylline, topical nitroglycerin, secukinumab) precludes attributing clinical improvement to any single intervention. Additionally, PD may stabilize spontaneously during its natural course, independent of treatment. Loss to follow-up precluded long-term outcome assessment and confirmation of sustained improvement.

Limitations also exist in the current body of evidence evaluated for this review. Most available studies consist of retrospective analyses, case reports, or cross-sectional studies that use variable and sometimes inconsistent diagnostic criteria for both conditions. Claim-based studies rely on administrative coding, which can misclassify relatively uncommon diagnoses such as PD, and short follow-up durations may miss conditions with more extended latency periods [6]. Aggregated diagnostic categories can also make it challenging to distinguish mechanistic differences between autoimmune and fibroproliferative processes. Additionally, publication bias likely favors studies showing positive associations, and the absence of prospective, longitudinal data makes it exceedingly difficult to establish temporal or causal links between systemic inflammation and localized fibrotic remodeling of the penile tunica albuginea. Due to these limitations, the relationships between immune dysregulation and fibrotic comorbidities across studies examining PD’s relationship with immune dysregulation remain unclear.

Future research should aim to address these gaps through well-designed prospective cohort studies that incorporate standardized phenotyping of PD and PsA and integrate molecular and genomic data. Tissue-level analyses of PD plaques for PsA-associated cytokine or immune signatures could clarify whether shared inflammatory pathways, such as IL-23/Th17 activation or TGF-β1-mediated fibrosis, drive both conditions. Genomic and transcriptomic profiling could further elucidate overlapping susceptibility loci or differentiate autoimmune-driven processes from primarily fibroproliferative mechanisms.

Finally, interventional studies assessing whether optimized control of PsA influences PD onset or progression would provide actionable clinical evidence to guide preventive strategies in at-risk populations. Such research may determine whether PD in select patients represents a localized fibrotic manifestation of broader systemic inflammatory disease or a separate fibroproliferative condition that occasionally coexists with autoimmune disorders.

## Conclusions

This case highlights a possible and exploratory association between uncontrolled PsA and the development of PD. Chronic systemic inflammation may contribute to localized fibrotic changes within the penis, primarily when psoriatic disease is not adequately controlled. The main clinical implications include maintaining therapeutic continuity for PsA through patient assistance programs and care coordination to prevent disease exacerbations; considering early urologic evaluation when patients with inflammatory arthritis report penile symptoms; and recognizing that social determinants can directly affect disease development and progression. Being aware of these potential associations and maintaining a high index of suspicion when patients with inflammatory arthritis present with urologic complications may help slow or prevent disease progression and address some of the barriers patients face when seeking care for PD.
